# Gold Inlays

**Published:** 1907-08-15

**Authors:** Charles F. Woodbury


### GOLD INLAYS.

CHARLES F. WOODBURY, D.D.S.

The subject of gold inlays has been receiving so much attention of late that it has seemed to the writer that many of the profession are in danger of going to extremes in their use.

Any dentist who no longer makes gold foil fillings in cavities that involve two surfaces, or who uses no gold foil at all is not, in my judgment, giving his patients the best service possible; provided always that he is able to make a good gold foil filling. A good gold foil filling made in a properly prepared cavity with a normal interproximal space and a contact point is the best protection against recurrence of decay of anything we have in our hands to-day, but a poor gold foil filling is the worst operation any dentist ever made for a patient. Therefore, when esthetic reasons do not forbid, always make a gold foil filling, if you are able, and the conditions do not contra-indicate it. If you do not feel absolute confidence in your ability to make a good gold foil filling in any cavity in front of the distal surface of the second molar you should at once "get busy" and improve your technique.

There are, however, conditions in which the use of gold inlays are indicated, and where, when properly constructed they serve a better purpose than anything we have at our command. The technical skill employed should be as accurate and painstaking as in any other operation we do, and the prevailing belief that the cement joint will cover a multitude of sins is a mistake and will only lead to grief. The fit of the inlay should be so close that when the margins are finished the joint between the inlay and the tooth will not be discernable through a ten diameter magnifying glass.

The cavity preparation should be as nearly as possible on the same lines as for foil fillings. The cavity margins should be laid where possible at the angles of the tooth and under the gum margins, as these are the positions which are the least susceptible to recurrence of decay. Special attention should be paid to the bucco-lingual extension at the gingival angles as it is very difficult to get as much extension at these vulnerable points as is necessary and have the cavity so shaped that the inlay will shed. My observation shows me that most inlay workers fail to get this extension. The gingival and pulpal walls should be made flat and at right angles to the long axis of the tooth and the axial walls as nearly at right angles to them as it is possible to have them and draw the inlay, as the nearer parallel these walls are the more firmly the inlay will be retained. The line angles should be square and definite.

Occlusal or incisal anchorage should always be used unless, as is occasionally advisable in the front teeth, a platinum and iridium post is used in the root canal. All undercuts should be filled with cement and if the axio-pulpal wall is badly decayed it should be restored. Mesio-occluso-distal cavities in molars should be made in two inlays, unless there is a great deal of loss of tooth structure, on account of difficulty of drawing the matrix without distortion. The margins of the cavity should be slightly beveled.

Gold inlays are indicated in the following places:

In large cavities in teeth of children.

In cavities in the teeth that are attacked by chronic interstitial gingivitis or pyorrhea.

In cavities in the teeth ot very nervous people or those the muscles of whose jaws are affected by cramps.

In cavities that are inaccessible either on account ot a small opening to the mouth or unusual position of the teeth. Accessibility should be the guide in many cavities whether to make a gold foil filling or an inlay.

In cavities the walls of which are too weak to stand the malleting necessary for gold foil and which are not so far gone as to necessitate crowns. This will include many teeth which in former years it would have been necessary to crown, but which in the hands ot the experienced inlay worker can be made to do years ot service and with a much more healthy environment than would have been the case had they been fitted with crowns.

In cavities proximating bridge work over which it is impossible to place a rubber dam.

It is possible tor very good inlays to be made in models of cement or amalgam. But in the hands of the ordinary operator I think it is apt to lead to inaccurate work and too much dependence is placed on the cement joint. Even in the hands of an experienced operator it has never seemed to me possible to obtain the absolutely invisible joint that can be made when working directly in the cavity. The writer in nine years' experience has used almost every method for making gold inlays that has appeared before the profession, but has discarded them all for the one now in use and the technique ot which follows in detail.

The cavity being prepared and the undercuts filled, a band matrix is adjusted around the tooth and wedged up in the interproximal space. The cavity is moistened and filled with base-plate gutta-percha. It is used as soft as possible and forced into the cavity with considerable pressure. The gutta-percha is chilled with compressed air or cold water, the band matrix and wedge taken out and this gutta-percha impression removed. This impression serves a double purpose. It proves the cavity preparation as the least undercut will draw the gutta-percha and be discernable. It is also used to assist in swaging the matrix in the cavity.

A generous sized piece of 1-1000 platinum is prepared, the lower end folded into the shape of an L, the lower stem or floor of the L being a little longer than the width of the interproximal space. This is to allow plenty of metal so that the platinum will not be drawn into the cavity so far that it will not cover the gingival margin. With a small piece of cotton or spunk the platinum is now forced into the axio-gingival angle. Additional pieces of cotton are packed in until the proximal space and cavity are filled level with the pulpal wall. The platinum is now bent over and worked into the occlusal step in the same manner. No account need be made of folds or tears unless the tears are on the margins in which case it is better to take another piece of platinum and start over. The platinum is taken out and trimmed to about 1-32 of an inch from the margins. It is again placed in the cavity and the gutta-percha impression forced into place with considerable pressure. The gutta-percha is taken out and the matrix carefully gone over with burnishers. The folds must be all burnished smooth and the matrix burnished into all the square angles of the cavity. A small piece of 23 gauge platinum wire is flattened on one end. The flattened part is bent in the shape of an L and soldered to the matrix, with pure gold, at the bottom of the occlusal step, the stem of the L being to protrude from the cavity over the axio-pulpal angle. This is used as a lifter in removing the matrix from the cavity.

The rubber dam is applied to the front teeth if possible although it is not absolutely necessary. The platinum matrix is placed in the cavity and a band matrix around the tooth and platinum. Sufficient annealed moss fibre gold is packed into the cavity to restore the contact and almost to restore the occlusion. Rather small pieces of gold should be used on the gingival, buccal and lingual margins and these should be quite well condensed with hand pressure, using large sized Royce pluggers. No effort should be made to thoroughly condense the body of the inlay, and quite large pieces of gold can be used, only sufficient density being obtained to prevent its distortion when being drawn from the cavity and during the subsequent flowing with plate gold. After the moss fibre gold has been packed in the platinum matrix, the band matrix is removed and all the margins carefully gone over and burnished, always burnishing from the gold on to the platinum and over the margin. The platinum wire lifter is now taken hold of securely with a pair of plyers and the inlay lifted out of the cavity. Care must be taken to remove it in a line parallel with the axial walls of the cavity, as the least rocking of the inlay during its removal is liable to distort the matrix and injure the adaptation at the margins.

The cavity surface is now covered with whiting and alcohol or some other anti-flux and the inlay is laid on a soldering block and enough S. S. W. 22 K plate is melted on it to thoroughly saturate the crystal gold and to restore the occlusal surface which was left a little scant when packing the crystal gold. Very little borax should be used, as it is unnecessary with such high carat of gold and an excess of borax is liable to run over on to the cavity side of the matrix carrying gold with it and thus spoil the work. The inlay is boiled in nitric acid to remove the whiting and borax. The contact point is shaped and polished and the gingival portion trimmed almost to the margin.

The inlay is now set, using for this purpose Ames' brown inlay cement of a reasonable consistency. With a wood stick or an instrument about 12 or 15 pounds of hand pressure and a succession of 10 pound blows from a hand mallet are applied to the inlay for a minute, as it takes that long where cement is mixed reasonably thick, for it to flow, leaving only the smallest possible quantity. The over hanging margins of the inlay are burnished from the gold over the cavity margins. The end of a wet cotton roll is placed on the inlay and the patient instructed to close the teeth on it firmly

If the rubber dam has been applied the clamp may be taken off and the patient instructed to close the teeth on the cotton and rubber. The inlay should now be covered with water and the patient told to maintain a firm pressure for about 15 or 20 minutes. It may now be finished with stones, discs and strips, always being careful that engine instruments rotate from the gold toward the margin. The final polish being given with pumice and whiting. I do not think it possible to finish and polish an inlay out of the mouth without taking off more than you should at one place and leaving overhanging margins at another.

If an inlay is to be made where it is necessary to restore both proximal and the whole occlusal surfaces of a tooth, the platinum matrix is fitted and one proximal side is filled with crystal gold a little more than level with the pulpal wall, letting the gold extend over the pulpal wall about half way to the opposite cavity. It is now taken out of the cavity, the matrix covered with whiting everywhere not covered by gold. Just enough gold is run in this to saturate it. It is boiled in nitric acid, placed in the cavity again and the proximal surface filled with crystal gold. This is now saturated with melted plate, again protecting the occlusal margins with whiting. There is much less danger of distortion in thus making one side at a time. The gingival portion of the inlay is now completed and the occlusal step about one-half filled.

A form suitable in size and shape to the occlusal surface to be restored is now struck up from a die-plate, using about 34 gauge pure gold plate. A ball of sticky wax is softened and placed inside this form. With the inlay in place in the cavity the soft wax is placed in it, the gold next the occluding teeth, and the patient instructed to close the teeth hard. The gold is now burnished into close adaptation to the matrix on the buccal and lingual surfaces and the patient again instructed to close the teeth and put the jaws through the various motions of chewing. The buccal and lingual portions are again reburnished. The inlay and cap are removed and invested, leaving one of the proximal surfaces exposed. The wax is burned out and the space between the inlay and cap filled with 22 K solder. The inlay is taken out of the investment, boiled in nitric acid, partially finished and set, leaving the final finishing to be done on the inlay while in the tooth after the cement has crystalized.

In conclusion let me repeat:

"Use inlays only where inlays are indicated."

"Use gold foil fillings where they are indicated."

"Use as much painstaking care with the inlays as with foil fillings."

"Don't depend too much on the cement."

"Have plenty of mechanical retention and always restore the interproximal space and contact point."

*Dentists Magazine.*

